# Saddlepoint inference for rank-based *k*-sample tests in clustered survival trials

**DOI:** 10.1038/s41598-026-44064-9

**Published:** 2026-04-02

**Authors:** Haidy A. Newer

**Affiliations:** Department of Mathematics, Faculty of Education, Ain Shams University, Cairo, 11511 Egypt

**Keywords:** Saddlepoint approximation, Cluster randomized trials, *k*-sample tests, Permutation inference, Clustered survival data, Ratio and product endpoints, Small-sample inference, Confidence intervals., Computational biology and bioinformatics, Mathematics and computing

## Abstract

Statistical inference for cluster randomized trials often involves complex derived endpoints, such as biomarker ratios or cumulative products, which significantly complicate the application of standard asymptotic theory. When the number of clusters is limited or intra-cluster dependence is pronounced, conventional nonparametric procedures relying on large-sample chi-squared approximations frequently break down, leading to inflated Type I error rates and inadequate confidence interval coverage. This paper develops a rigorous, unified framework to circumvent these inferential failures by constructing a rank-based *k*-sample test statistic specifically tailored for clustered, right-censored survival data. We derive a multivariate saddlepoint approximation for the exact permutation distribution under an urn randomization scheme, providing a solution that yields higher-order precision. This analytical strategy recovers the accuracy of computationally exhaustive Monte Carlo resampling at a fraction of the numerical cost. Furthermore, the efficiency of the saddlepoint approach enables the practical construction of nonparametric confidence intervals for relative treatment effects via test inversion. Extensive simulations demonstrate that our proposed method preserves nominal significance levels in the small-cluster regimes where standard techniques prove unreliable. The practical utility of the methodology is further evidenced through the analysis of three multi-center clinical trials-concerning leukemia, vision loss, and periodontitis-where the saddlepoint approximation provides robust and trustworthy inferential conclusions in borderline cases that would otherwise yield conflicting results.

## Introduction

Evaluating multiple interventions within a single experimental framework is central to the rigorous advancement of clinical and epidemiological research. While traditional statistical methodologies are well-equipped to handle simple univariate outcomes, modern scientific inquiry is increasingly characterized by the use of derived quantities-such as biomarker ratios or the cumulative products of longitudinal measurements-as primary endpoints. Testing for distributional disparities across multiple groups using such derived data presents a formidable inferential challenge, particularly when the underlying probability distributions are non-normal or entirely unknown.

This complexity is compounded by the pragmatic necessities of experimental design. A significant portion of contemporary studies in public health and medicine utilize cluster randomized designs, where randomization is performed at the level of the unit (e.g., clinics, schools, or geographical communities) rather than the individual^1^. Although this approach facilitates logistical feasibility and mitigates treatment contamination, it inevitably introduces intra-cluster correlation. This dependency structure invalidates the independence assumptions fundamental to standard rank-based tests. When the endpoint of interest is a time-to-event, the resulting clustered survival data require specialized analytical treatment. A foundational framework for this setting was proposed by^2^, who developed a quadratic test statistic based on cluster-level scores to account for the internal correlation. However, the inferential validity of this statistic typically rests upon large-sample asymptotic theory-specifically, convergence to a chi-squared distribution. In practice, clinical trials often involve a limited number of clusters, a scenario where asymptotic approximations frequently become unreliable, leading to inflated Type I error rates or a substantial loss of power. In these finite-sample settings, the exact permutation distribution remains the gold standard for inference; however, its direct enumeration is computationally prohibitive for all but the most trivial datasets.

The validity of any permutation-based inference is inherently tied to the physical randomization process employed in the study. The urn randomized design serves as a theoretically transparent and elegant mechanism for implementing complete randomization. By conceptually filling an urn with balls representing treatment assignments and drawing them without replacement as clusters are enrolled, this scheme ensures that target sample sizes for each treatment arm are met with absolute precision^3^. This physical process defines the discrete permutation space that serves as the basis for our proposed inferential framework.

Saddlepoint approximations offer a sophisticated and powerful alternative to the computational brute force of Monte Carlo simulations and the potential inaccuracies of first-order asymptotics. By utilizing the entire cumulant generating function rather than just the first two moments, this technique provides analytical formulas for *p*-values that remain remarkably accurate even in the extreme tails of the distribution. The utility of saddlepoint-based inference for permutation tests with censored data was established by^4^ in the context of trend tests. Subsequent research has affirmed the robustness of this approach across varied experimental landscapes, including basic random allocation^5^, stratified designs^6^, and adaptive schemes such as block truncated binomial and Efron’s biased-coin designs^7–12^.

Despite these advancements, a unified framework that extends saddlepoint precision to *k*-sample tests specifically for *ratios and products* in the presence of clustering has remained an unexplored gap in the literature. This paper fills that critical void. We synthesize these distinct research trajectories by adapting *k*-sample statistics for clustered data to handle derived ratio and log-product observations, utilizing the robust scoring methodology of^13^. We then derive a multivariate saddlepoint approximation tailored to the exact permutation distribution generated by urn randomization. This methodology not only yields high-precision *p*-values but also, through its computational efficiency, enables the practical inversion of the test to construct non-parametric confidence intervals for relative treatment effects.

The work is organized as follows. Section The k-sample test for clustered data outlines the methodological foundations, including the urn design, the construction of cluster-level scores for complex endpoints, and the formulation of the global quadratic statistic. Section Saddlepoint approximation for the permutation distribution details our primary theoretical contribution: the derivation of the multivariate saddlepoint approximation. Section Confidence intervals by test inversion demonstrates the extension of this framework to confidence interval estimation via test inversion. In Section Simulation algorithm and design, we present extensive simulation evidence to validate the accuracy of our approach regarding error control and interval coverage. Section Application to real-data clinical scenarios illustrates the practical necessity of the method through the analysis of real-world multi-center clinical datasets. Finally, Section Conclusion provides a summary of our findings and discusses prospective avenues for future research.

## The *k*-sample test for clustered data

This section formalizes a robust non-parametric framework for *k*-sample comparisons when outcomes are represented as complex, cluster-level derivations. Our methodological approach follows a rigorous logical progression: beginning with the foundational principles of randomization, moving through the distillation of individual-level data, and culminating in the construction of a global quadratic test statistic. We first define the urn randomization scheme, which establishes the theoretical substrate for valid permutation inference. We then introduce a data-transformation strategy that converts intricate, potentially censored observations into a singular, representative score for each cluster. Finally, these scores are aggregated into a quadratic form to test the null hypothesis of distributional invariance across treatment arms.

### The urn randomized design for clusters

The validity of any permutation-based inferential procedure is fundamentally tethered to the physical mechanism of treatment allocation. To ensure that our statistical analysis strictly mirrors the experimental design, our framework is anchored by the urn randomized design-a theoretically transparent method for implementing a complete randomized trial at the cluster level^14^. This model ensures that a pre-specified number of clusters, $N_j$, is allocated to each treatment group *j* with deterministic certainty^3^. The formal protocol for assigning *N* independent clusters to *k* distinct treatment groups is defined as follows:

1. *Allocation specification* Target sample sizes for each group are predetermined as $N_1, N_2, \ldots , N_k$, such that $\sum _{j=1}^{k} N_j = N$.
2. *Urn composition* A conceptual urn is populated with *N* balls, where $N_1$ balls are indexed for treatment 1, $N_2$ for treatment 2, and so forth, until $N_k$ for treatment *k*.
3. *Sequential assignment* For each enrolling cluster, a ball is drawn from the urn *without replacement*. The cluster is then assigned the specific treatment designated by the drawn ball, a process that continues until the urn is exhausted.

This procedure represents a discrete application of a complete randomized design (CRD), where every possible combinatorial allocation of clusters into groups of the specified sizes is assigned an equal probability^15^. The CRD serves as the foundational architecture for cluster randomized trials, particularly in the absence of compelling evidence to balance specific cluster-level covariates via stratification or blocking^1^.

Crucially, this design precisely delineates the reference distribution for our test statistic under the null hypothesis of no treatment effect. In this permutation framework, we condition the inference on the observed set of cluster scores, treating them as fixed realizations. The stochasticity arises solely from the assignment mechanism. Under $H_0$, the treatment labels are randomly reassigned across these fixed scores. The cardinality of this permutation space is defined by the multinomial coefficient, $\frac{N!}{N_1! N_2! \cdots N_k!}$^16^. Consequently, our inferential task is to quantify the extremity of the observed statistic relative to the distribution of all potential realizations across this finite space.

### Derived time-to-event endpoints

A pervasive challenge in modern biostatistics is the analysis of primary endpoints that are not simple measurements but complex quantities synthesized from multiple raw variables. Our framework circumvents the distribution-related complexities of these endpoints by reframing them-specifically ratios and products-as generalized *time-to-event* data. This conceptualization, where “time” represents a positive, continuous measure of accumulation until a threshold event, is a well-supported principle in survival analysis^17^. This abstraction provides a unified analytical trajectory: once the derived variable $T_i$ is constructed, it can be subjected to robust, rank-based permutation tests regardless of its original dimensionality.

Let the raw data for subject *i* be a vector of positive measurements $(X_{i,1}, X_{i,2}, \ldots , X_{i,p})$ used to compute the latent event time $T_i$. Under this generalized view, the standard right-censoring framework applies naturally. If a subject is lost to follow-up or the observation window closes prior to event occurrence, the data are censored. Formally, each subject is represented by the pair $(T_i^{\text {obs}}, \delta _i)$, where $T_i^{\text {obs}} = \min (T_i, C_i)$ is the observed time, and $\delta _i = I(T_i \le C_i)$ is the event indicator. We focus on two specific derived endpoints:

Case 1: The ratio as a time-to-event. In contexts where the relationship between two concentrations is the metric of interest (e.g., biomarker ratios), we define the event “time” for subject *i* as:

$$T_i = R_i = \frac{X_{i,1}}{X_{i,2}}.$$

The statistical test evaluates the survival distributions of these ratios across the *k* groups, determining if one arm reaches critical ratio values systematically “sooner” (i.e., at a lower magnitude) than others.

Case 2: The product as a time-to-event. Alternatively, the endpoint may reflect a cumulative or multiplicative pathway effect. Here, the event time is the product of the measurements:

$$T_i = P_i = \prod _{p=1}^{P} X_{i,p}.$$

To stabilize variance and convert the multiplicative scale into an additive one, we apply a logarithmic transformation, $\log (T_i)$. This recasts the comparison as a test for location-scale shifts in the distribution of the log-transformed products.

This transformation into a standardized survival format is the methodological cornerstone of our approach. It renders the subsequent inference *distribution-free* with respect to $T_i$, bypassing the often-intractable problem of deriving exact probability density functions for ratios or products of random variables. Instead, the test relies purely on the relative ranks of the observed times.

### Cluster scoring via weighted log-rank statistics

To account for censoring within a clustered architecture, we employ a two-stage scoring protocol. The first stage handles individual-level censoring by assigning a “censoring-adjusted weighted rank” to each subject using the class of weighted log-rank statistics^18^. We pool all *M* subjects (where $M = \sum _{c=1}^N n_c$) across all clusters and groups. For this pooled sample, we compute an individual score $w_i$ based on the classic observed-minus-expected paradigm:

2.1 $$\begin{aligned} w_i = W(T_i^{\text {obs}})\delta _i - \sum _{j: t_j \le T_i^{\text {obs}}} W(t_j)\frac{d_j}{n_j}, \end{aligned}$$

where the summation covers unique event times $t_j$ up to $T_i^{\text {obs}}$, $d_j$ represents events at $t_j$, $n_j$ is the risk-set size, and *W*(*t*) is a predefined weight function. As demonstrated by^2^, this formulation unifies various non-parametric tests. By modulating *W*(*t*), the test can be optimized for specific survival profiles:

- *Log-rank test* $W(t) = 1$, optimal for proportional hazards^19^.
- *Gehan-Wilcoxon test* $W(t) = n_t$, emphasizing early event times and powerful under non-constant hazard ratios^20^.
- *Prentice-Wilcoxon test* $W(t) = \hat{S}(t^-)$, prioritizing early failures through the estimated survival function^13^.

The second stage resolves the clustered nature of the data by aggregating individual scores into the randomization units. This step is vital for capturing the intra-cluster correlation. The total score for cluster *c*, denoted $C_c$, is the sum of its constituent individual scores:

2.2 $$\begin{aligned} C_c = \sum _{i \in \text {cluster } c} w_i. \end{aligned}$$

The resulting vector $\{C_1, C_2, \ldots , C_{N}\}$ distills the complex dataset into a robust, cluster-level representation prepared for *k*-sample permutation analysis.

### The quadratic test statistic for *k*-sample comparison

Using the robust scores $\{C_c\}$, we construct a global test statistic to detect significant disparities across the *k* treatment arms. We first define a $(k-1)$-dimensional test vector, $\mathbf{W}$, where each component $W_j$ represents the total score for the *j*-th treatment group:

$$W_j = \sum _{c \in G_j} C_c,$$

where $G_j$ denotes the indices of clusters assigned to group *j*. To ensure the statistic is valid and scale-invariant, we standardize $\mathbf{W}$ using the exact variance-covariance matrix, $\mathbf {\Sigma }$, derived from the combinatorial properties of the urn design. This is equivalent to finite population sampling without replacement^14^, with terms defined as:

2.3 $$\begin{aligned} \text {Var}(W_j)&= \frac{N_j(N - N_j)}{N - 1} S_C^2, \end{aligned}$$

2.4 $$\begin{aligned} \text {Cov}(W_j, W_l)&= \frac{-N_j N_l}{N - 1} S_C^2, \quad \text {for } j \ne l , \end{aligned}$$

where $S_C^2 = \frac{1}{N} \sum _{c=1}^{N} C_c^2$ is the variance of the cluster scores (the mean being zero by definition).

The final quadratic test statistic, *Q*, encapsulates the squared standardized distance of the observed group effects from the null expectation:

2.5 $$\begin{aligned} Q = \mathbf{W}^T \mathbf {\Sigma }^{-1} \mathbf{W}. \end{aligned}$$

While *Q* is asymptotically $\chi ^2_{k-1}$ distributed in large samples^2^, this approximation often proves unreliable in small-cluster settings. The primary contribution of this work is to sidestep this asymptotic dependence by calculating *p*-values directly from the exact permutation distribution via the multivariate saddlepoint approximation detailed in the following section.

## Saddlepoint approximation for the permutation distribution

Calculating the exact permutation distribution for a *k*-sample test statistic in the presence of clustered or censored data is frequently computationally intractable. The sheer volume of potential randomizations scales factorially with the number of clusters, rendering direct enumeration impossible. Furthermore, standard asymptotic approximations, such as those based on chi-squared limits, often fail to provide reliable inference when cluster counts are low or when outcomes exhibit significant correlation. To circumvent these limitations, we employ the *saddlepoint method*-an elegant analytical technique that leverages the cumulant generating function (CGF) of the test statistic to reconstruct its distribution with high precision. This approach preserves the near-exact nature of permutation inference while bypassing the exhaustive computational burden associated with it^21–23^.

Permutation structure and score representation: The analytical foundation of our test is the urn randomization scheme described in Section The k-sample test for clustered data. This design distills the complex, multi-dimensional data from each of the *N* clusters into a single scalar score, $C_c$. Under the null hypothesis of no treatment effect, the assignment of these *N* clusters to the *k* treatment arms is random, generating a finite permutation space $\Pi$ with cardinality $N!/(N_1!\cdots N_k!)$. To facilitate the derivation, we first assemble the full set of cluster scores into a centered vector:

3.1 $$\begin{aligned} \mathbf{C} = (C_1, \ldots , C_N)^{\top }. \end{aligned}$$

The allocation of clusters to treatment groups is encoded in a contrast matrix $\mathbf{A}$ of dimension $(k-1)\times N$, where each row defines a specific contrast between treatment indicators such that $\mathbf{A}\mathbf{1}_N = \mathbf{0}$. The test vector $\mathbf{W}$, representing the aggregated scores for the first $k-1$ groups, is derived via the linear transformation:

3.2 $$\begin{aligned} \mathbf{W} = \mathbf{A}\mathbf{C}, \qquad \mathbf{W} \in \mathbb {R}^{k-1}. \end{aligned}$$

Under the null hypothesis, $\mathbf{W}$ follows a discrete permutation distribution induced by the random reassignment of cluster scores. Our global test statistic is a quadratic form measuring the standardized divergence of the observed treatment effects from the null expectation:

3.3 $$\begin{aligned} Q = \mathbf{W}^{\top }\boldsymbol{\Sigma }^{-1}\mathbf{W}, \end{aligned}$$

where $\boldsymbol{\Sigma }$ denotes the exact covariance matrix of $\mathbf{W}$ under the null permutation distribution, as defined in Eqs. (2.3) and (2.4). This formulation generalizes the classical rank-based *k*-sample statistic, accommodating complex nonlinear data transformations such as ratios or products within the vector $\mathbf{W}$.

### The multivariate saddlepoint approximation

The core engine of the saddlepoint method is the CGF of the random vector $\mathbf{W}$. Given the urn randomization scheme, which implies a uniform probability mass over the permutation space $\Pi$, the CGF is formally defined as:

3.4 $$\begin{aligned} K(\mathbf{s}) = \log \left( \frac{1}{|\Pi |} \sum _{\pi \in \Pi } \exp \left( \mathbf{s}^\top \mathbf{W}_\pi \right) \right) , \end{aligned}$$

where $\mathbf{W}_\pi$ is the realization of the test statistic under a specific permutation $\pi$, and $\mathbf{s} \in \mathbb {R}^{k-1}$ represents the vector of canonical parameters.

A critical property for the existence of the saddlepoint is the convexity of the CGF. Provided the cluster scores are non-degenerate and the number of clusters *N* exceeds the number of groups *k*, the covariance matrix $\boldsymbol{\Sigma }$ is positive definite. This guarantees that $K(\mathbf{s})$ is strictly convex over its domain, ensuring that the gradient equation $\nabla K(\hat{\mathbf{s}}) = \mathbf{w}$ has a unique real solution $\hat{\mathbf{s}}$ for any observed $\mathbf{w}$ within the convex hull of the permutation support.

Solving this gradient equation for the saddlepoint $\hat{\mathbf{s}}$ requires a numerical root-finding procedure, such as the Newton-Raphson method. Unlike the computationally expensive Monte Carlo simulations needed for exact permutation tests, this numerical optimization is highly efficient, typically converging in milliseconds even for large datasets. The saddlepoint $\hat{\mathbf{s}}$ identifies the specific exponential tilting of the distribution that centers its mean at the observed value $\mathbf{w}$. By performing a Taylor expansion of $K(\mathbf{s})$ around $\hat{\mathbf{s}}$ and applying the multivariate Laplace approximation, we derive the saddlepoint density for $\mathbf{W}$:

3.5 $$\begin{aligned} f_{\text {SP}}(\mathbf{w}) = (2\pi )^{-\frac{k-1}{2}}|\det K''(\hat{\mathbf{s}})|^{-\frac{1}{2}} \exp \!\left\{ K(\hat{\mathbf{s}}) - \hat{\mathbf{s}}^{\top }\mathbf{w}\right\} , \end{aligned}$$

where $K''(\hat{\mathbf{s}})$ is the Hessian matrix of second derivatives of the CGF evaluated at $\hat{\mathbf{s}}$^21,22^. This approximation achieves *exponential accuracy*, with relative errors diminishing faster than any polynomial order of the sample size *N*.

### Saddlepoint approximation for the quadratic form *Q*

While the multivariate density describes the behavior of the vector $\mathbf{W}$, our inference relies on the scalar quadratic statistic *Q*. To obtain the distribution of *Q*, we effectively integrate the multivariate saddlepoint density $f_{\text {SP}}(\mathbf{w})$ over the manifold defined by the quadratic constraint:

3.6 $$\begin{aligned} f_Q(q) = \int _{\{\mathbf{w} : \mathbf{w}^\top \boldsymbol{\Sigma }^{-1} \mathbf{w} = q\}} f_{\text {SP}}(\mathbf{w}) \, d\mathbf{w}. \end{aligned}$$

The transition from the vector field to the scalar density is achieved analytically by applying a specialized univariate Laplace approximation to this surface integral. This yields the explicit scalar density:

3.7 $$\begin{aligned} f_{\text {SP}}(q) = (2\pi )^{-1/2}[K_Q''(\hat{t})]^{-1/2} \exp \!\{K_Q(\hat{t}) - \hat{t}q\}, \end{aligned}$$

where $K_Q(t) = \log E[\exp (tQ)]$ represents the CGF of the scalar statistic *Q*, and $\hat{t}$ is the unique saddlepoint solving $K_Q'(\hat{t}) = q$^23^. This formula provides a direct, highly accurate analytical route to the permutation distribution of *Q*, eliminating the need for simulation.

Solving for the saddlepoint $\hat{t}$ (analogous to $\hat{\mathbf{s}}$) involves a standard numerical root-finding algorithm. We utilize the Newton-Raphson method, leveraging the analytical Hessian $K''(\mathbf{s})$. Given the strict convexity of the CGF, this algorithm is globally convergent and stable, typically reaching the solution within 5 to 10 iterations. In rare instances of near-singularity-such as datasets with extremely small sample sizes or heavy ties-standard Levenberg-Marquardt regularization can be applied to ensure robust convergence.

### Tail probability and the mid-*p* value

With the CGF of *Q* established, we approximate the upper-tail probability using the renowned *Lugannani–Rice* expansion^24,25^:

3.8 $$\begin{aligned} P(Q \ge Q_{\text {obs}}) \approx 1 - \Phi (r) + \phi (r)\!\left( \frac{1}{r} - \frac{1}{u}\right) , \end{aligned}$$

where the standardized terms *r* and *u* are defined as:

3.9 $$\begin{aligned} r = \operatorname {sign}(\hat{t})\sqrt{2\{\hat{t}Q_{\text {obs}} - K_Q(\hat{t})\}}, \qquad u = \hat{t}\sqrt{K_Q''(\hat{t})}, \end{aligned}$$

and $\Phi (\cdot )$ and $\phi (\cdot )$ denote the standard normal cumulative distribution and density functions, respectively. This expansion provides third-order accuracy, with a relative error of $O(N^{-3/2})$.

A final refinement addresses the inherent discreteness of the permutation distribution. Standard *p*-values for discrete statistics can be overly conservative. To mitigate this, we adopt the *mid-*p value:

3.10 $$\begin{aligned} P_{\text {mid}}(Q_{\text {obs}}) = P(Q > Q_{\text {obs}}) + \tfrac{1}{2}P(Q = Q_{\text {obs}}). \end{aligned}$$

In Monte Carlo frameworks, the mid-*p* value yields near-exact results even with limited clusters^26–28^. This adjustment effectively corrects for the conservatism of discrete tests, ensuring that confidence intervals derived via test inversion achieve their nominal coverage.

In summary, the saddlepoint approximation for *Q* serves as a powerful analytical bridge, connecting the complex multivariate permutation structure of $\mathbf{W}$ to an accurate tail probability for the test statistic. It combines the theoretical rigor of exact permutation tests with the computational tractability of asymptotic methods. Compared to Monte Carlo resampling, this saddlepoint-based inference is deterministic, computationally efficient, and robust to variations in cluster size and censoring, making it an ideal tool for the complex *k*-sample designs addressed in this work.

## Confidence intervals by test inversion

A compelling practical advantage of our precise saddlepoint approximation is its capacity to generate robust non-parametric confidence intervals via test inversion. This approach exploits the fundamental duality between hypothesis testing and interval estimation: a $(1-\alpha )$ confidence region is defined as the set of all parameter values that cannot be rejected as the null hypothesis at the $\alpha$ significance level^29^. Because the validity of such intervals hinges directly on the accuracy of the underlying *p*-values, the superior precision of the saddlepoint method-particularly in the tails of the distribution-makes it uniquely suited for this task.

We apply this methodology to construct confidence intervals for relative treatment effects on complex derived endpoints. Conceptually, the procedure identifies the range of effect sizes for which the treatment groups, once mathematically adjusted for the hypothesized effect, become statistically indistinguishable.

Confidence intervals for relative ratio effects: For ratio-based endpoints, treatment effects are naturally conceptualized within a multiplicative framework. Let $R_j$ denote the random variable representing the ratio for a subject in group *j*, and let $R_0$ represent the baseline distribution. The treatment effect is modeled as a scale transformation:

$$\psi (R_j) \sim \psi (\theta _j \cdot R_0).$$

Here, $\theta _j$ quantifies the multiplicative effect of treatment *j* relative to the baseline, with the null hypothesis of no effect corresponding to $\theta _j = 1$ for all *j*. To construct the confidence region for the vector of effects $(\theta _1, \dots , \theta _k)$, we invert the *k*-sample permutation test as follows:

1. Define a grid of plausible candidate values for the effect vector $(\theta _1, \dots , \theta _k)$.
2. For each candidate vector on the grid, transform the original observed data. Specifically, if $T_{ij}$ is the observed ratio for subject *i* in group *j*, the adjusted observation under the null hypothesis is computed as $T_{ij}^* = T_{ij} / \theta _j$.
3. Re-evaluate the *k*-sample test statistic *Q* using these adjusted scores and compute the corresponding saddlepoint *p*-value.
4. The $(1-\alpha )$ confidence region consists of all vectors $(\theta _1, \dots , \theta _k)$ for which the resulting *p*-value exceeds $\alpha$.

Confidence intervals for relative product effects: For product-based endpoints, analysis is most robust on the logarithmic scale, converting the relationship into an additive model. Let $L_j = \log (P_j)$ represent the log-transformed product for a subject in group *j*. The model is specified as a location shift:

$$\varphi (L_j) \sim \varphi (L_0 + \Delta _j),$$

where $\Delta _j$ represents the additive shift parameter for group *j*. The inversion procedure parallels the ratio case but utilizes an additive adjustment:

1. Establish a grid of potential values for the additive shift vector $(\Delta _1, \dots , \Delta _k)$.
2. Generate adjusted data for each hypothesized vector. If $L_{ij}$ is the log-product for subject *i* in group *j*, the adjusted value is $L_{ij}^* = L_{ij} - \Delta _j$.
3. Recalculate the test statistic *Q* and its saddlepoint *p*-value based on the adjusted data.
4. Define the confidence region for $(\Delta _1, \dots , \Delta _k)$ as the set of all vectors yielding a *p*-value greater than $\alpha$.

Finally, confidence intervals for the original multiplicative scale can be obtained by exponentiating the endpoints of the intervals derived for the additive shifts $\Delta _j$.

*An illustrative example: The Cox frailty framework* The test inversion principle is a versatile tool that extends beyond non-parametric settings. To illustrate the complexities it can resolve, we consider the parametric Cox shared frailty model for clustered survival data. The hazard function for subject *i* in cluster *j* is defined as^30^:

$$\lambda _{ij}(t | u_j) = u_j \lambda _0(t) \exp (\boldsymbol{\beta }^{\top }\mathbf{X}_{ij}),$$

where $u_j$ is the shared frailty term inducing intra-cluster correlation. While our primary focus is testing the vector of treatment coefficients (i.e., $H_0: \boldsymbol{\beta } = \mathbf{0}$), this model presents a unique inferential challenge regarding the frailty variance, $\sigma _u^2 = \text {Var}(u_j)$, which quantifies the magnitude of clustering.

Testing for the absence of clustering corresponds to the null hypothesis $H_0: \sigma _u^2 = 0$. Since variance must be non-negative, the null value lies on the boundary of the allowable parameter space. This violation of standard regularity conditions renders Wald-based confidence intervals unreliable, often resulting in physically impossible negative lower bounds^31^. A rigorous solution involves inverting the likelihood ratio test (LRT). As demonstrated by^32^, the asymptotic null distribution of the LRT statistic in this boundary case is a mixture of chi-squared distributions ($\frac{1}{2}\chi ^2_0 + \frac{1}{2}\chi ^2_1$) rather than a standard $\chi ^2_1$. By correctly inverting the LRT based on this mixture distribution, one can derive valid confidence intervals for the frailty variance, illustrating the broader power of test inversion strategies in statistical inference.

## Simulation algorithm and design

To rigorously evaluate the operating characteristics of the proposed saddlepoint approximation, we designed a comprehensive simulation study. The protocol was structured to replicate the complex conditions frequently encountered in cluster randomized trials, specifically addressing multi-arm comparisons, heterogeneity in cluster sizes, and varying degrees of intra-cluster correlation.

### Data generation framework

Our simulation engine is built upon the shared gamma frailty model, a standard and flexible approach for generating correlated survival data^30^. The conditional hazard for an individual $i$ within a cluster $j$, given the cluster-specific frailty $u_j$, is defined by the Cox proportional hazards model:

$$\lambda _{ij}(t | u_j) = u_j \lambda _0(t) \exp (\boldsymbol{\beta }^{\top }\mathbf{X}_{ij}).$$

The primary objective of this study was to assess the control of Type I error rates. Consequently, all data were simulated under the global null hypothesis ($H_0$) of no treatment effect ($\boldsymbol{\beta } = \mathbf{0}$). Under this condition, the model simplifies to $\lambda _{ij}(t | u_j) = u_j \lambda _0(t)$, implying that all variation in outcomes is driven solely by the baseline hazard and the shared frailty term.

The complete, step-by-step procedure for generating a single dataset is outlined below:

1. Scenario specification and frailty generation: First, a specific simulation scenario is selected from Table 1, which defines the key parameters: the number of treatment groups (*k*), the total number of clusters (*N*), the distribution of cluster sizes (*m*), the intra-cluster correlation parameter ($\theta$), and the target censoring proportion. For each of the *N* clusters, a random frailty value $u_j$ is drawn from a gamma distribution with mean 1 and variance $\theta$ (specifically, Gamma(shape=$1/\theta$, scale=$\theta$)). In scenarios specifying independence ($\theta = 0$), all frailty terms $u_j$ are fixed to 1.
2. Latent event time generation: This step defines the underlying outcome variable. We simulated two distinct types of derived endpoints to assess the versatility of the method.
  For derived ratio endpoints: When the outcome is defined as a ratio, we first simulate its constituent components. For each subject *i* in cluster *j*, two independent positive variables, $X_{i,1}$ and $X_{i,2}$, are generated from a baseline distribution (either Weibull or exponential). The intra-cluster correlation is induced by using the frailty $u_j$ to scale the rate parameter of the exponential or the scale parameter of the Weibull distribution. The latent event time for the subject is then computed as the ratio:
    $$\begin{aligned} T_{ij} = R_{ij} = \frac{X_{i,1}}{X_{i,2}}. \end{aligned}$$
  For derived product endpoints: The process is analogous for product-based endpoints. A set of *p* underlying positive variables ($X_{i,1}, \dots , X_{i,p}$) are generated from a baseline distribution (either Weibull or exponential) for each subject, with their parameters modified by the shared frailty $u_j$. The latent event time is calculated as their cumulative product:
    $$\begin{aligned} T_{ij} = P_{ij} = \prod _{l=1}^{p} X_{i,l}. \end{aligned}$$
    To improve numerical stability and align with standard linear modeling assumptions, this product is subsequently log-transformed.
3. Censoring and final data observation: The latent event time $T_{ij}$ generated from either process (A or B) is subjected to independent right-censoring. A censoring time $C_{ij}$ is generated for each subject from an exponential distribution, with its rate parameter calibrated to achieve the target censoring proportion for the scenario (e.g., 20% or 40%). The final observed data for each subject consists of the pair $(T^{obs}_{ij}, \delta _{ij})$, defined as:
  $$T^{obs}_{ij} = \min (T_{ij}, C_{ij}) \quad \text {and} \quad \delta _{ij} = I(T_{ij} \le C_{ij}).$$

Simulation scenarios: We specified 20 challenging scenarios, detailed in Table 1, to stress-test the inferential methods across a broad spectrum of experimental conditions. The parameters were systematically varied to cover:

- Number of treatment groups k: Multi-arm clinical trials involving 5 and 6 distinct groups.
- Number of clusters N: A wide range spanning small (25–30), moderate (50–75), and large (100–180) sample sizes.
- Cluster sizes: Both balanced designs with uniform cluster sizes and unbalanced designs where sizes were drawn from a discrete uniform distribution (U[a,b]).
- Intra-cluster correlation:  ($\theta$): Low (0.25), moderate (0.50), and high (0.75) levels of dependency. To isolate the impact of clustering structure from correlation magnitude, a subset of simulations was conducted with $\theta$ set to zero.
- Censoring rates: Moderate (20%) and high (40%) censoring proportions.

Each unique parameter configuration was replicated 10,000 times to ensure the stability and precision of the estimated Type I error rates.

**Table 1:** Systematic configuration of the 20 simulation scenarios, categorized by the specific test statistic framework and experimental design parameters.

Scenario	Groups (*k*)	Clusters (*N*)	Cluster size (*m*)	Correlation ($\theta$)	Censoring (%)
Log-rank Test Framework (Scenarios 1–6)					
1	5	25	Balanced (5)	0.25	$\sim$20
2	5	30	Unbalanced (*U*[3, 7])	0.50	$\sim$40
3	5	50	Balanced (10)	0.75	$\sim$20
4	5	75	Unbalanced (*U*[5, 15])	0.25	$\sim$40
5	5	100	Balanced (15)	0.50	$\sim$20
6	5	100	Unbalanced (*U*[10, 20])	0.00	$\sim$40
Gehan–Wilcoxon Test Framework (Scenarios 7–13)					
7	6	30	Balanced (5)	0.50	$\sim$20
8	6	30	Unbalanced (*U*[3, 7])	0.75	$\sim$40
9	6	60	Balanced (10)	0.25	$\sim$20
10	6	75	Unbalanced (*U*[5, 15])	0.50	$\sim$40
11	6	120	Balanced (15)	0.25	$\sim$20
12	6	180	Unbalanced (*U*[10, 20])	0.75	$\sim$40
13	6	180	Balanced (20)	0.00	$\sim$20
Prentice–Wilcoxon Test Framework (Scenarios 14–20)					
14	5	25	Unbalanced (*U*[3, 7])	0.75	$\sim$20
15	5	50	Balanced (5)	0.25	$\sim$40
16	5	75	Balanced (10)	0.50	$\sim$20
17	6	60	Unbalanced (*U*[5, 15])	0.75	$\sim$40
18	6	120	Balanced (10)	0.50	$\sim$20
19	5	50	Unbalanced (*U*[5, 15])	0.00	$\sim$20
20	6	60	Balanced (5)	0.00	$\sim$40

Randomization and performance evaluation: Once a dataset was generated, the $N$ clusters were allocated to the $k$ treatment groups via an urn randomization procedure. This method guarantees that the pre-specified number of clusters for each arm is met exactly^3^. Following randomization, we tested the global null hypothesis of no difference between groups using the quadratic test statistic $Q$. The statistical significance of this statistic was assessed using three distinct methodologies:

- Standard Chi-Squared p-value: The conventional large-sample approximation, which compares the observed statistic to the upper tail of a $\chi ^2_{k-1}$ distribution.
- Saddlepoint p-value: The proposed analytical method, computed using the highly accurate Lugannani-Rice formula derived from the cumulant generating function of the permutation distribution.
- Mid-p-value A near-exact benchmark, serving as the gold standard. This value was estimated from 10,000 random permutations of the cluster scores. To adjust for the discrete nature of the permutation distribution, the mid-p-value was calculated as:
  $$\begin{aligned} P_{\text {mid}}(Q_{\text {obs}}) \approx \frac{\#\{Q_{\text {perm}} > Q_{\text {obs}}\} + 0.5 \times \#\{Q_{\text {perm}} = Q_{\text {obs}}\}}{10,000}. \end{aligned}$$

### Simulation results and discussion

We evaluated the inferential validity of the proposed saddlepoint method against the standard chi-squared approximation and the near-exact permutation mid-$p$ value. Performance was assessed based on empirical Type I error rates across 10,000 replications per scenario, with a nominal significance threshold of $\alpha = 0.05$. The results, detailed in Tables 2 and 3, demonstrate the following key findings:

1. Performance for ratio endpoints (Table 2):
  The proposed saddlepoint approximation demonstrated remarkable accuracy, tracking the gold-standard mid-$p$ value almost identically. Across all distributional assumptions (Weibull and Exponential), the saddlepoint method maintained Type I error rates tightly clustered around 0.05, even in scenarios characterized by high intra-cluster correlation ($\theta = 0.75$) and limited sample sizes.
  Conversely, the chi-squared test proved unreliable in small-to-moderate sample settings with non-negligible correlation. The most severe inflation occurred in Scenario 8 ($k=6, N=30, \theta =0.75$), where the asymptotic test rejected the null hypothesis in over 13% of simulations-more than double the nominal rate. This confirms that standard large-sample theory fails to account for the reduction in effective sample size caused by clustering.
2. Performance for log-product endpoints (Table 3):
  The inferential patterns observed for log-product endpoints mirror those of the ratio analyses. The saddlepoint approximation retained its robustness, providing valid error control regardless of the underlying data structure.
  While the logarithmic transformation is generally effective for variance stabilization, it did not rectify the liberal bias of the chi-squared statistic. In settings with few clusters, the asymptotic test continued to yield unacceptably high error rates, reinforcing the necessity for higher-order approximations in this context.

In summary, the multivariate saddlepoint approximation successfully bridges the gap between computationally prohibitive exact methods and often-unreliable asymptotic limits. By delivering permutation-level accuracy at a fraction of the computational cost, it offers a robust solution for valid inference in cluster randomized trials with complex endpoints.

**Table 2:** Empirical Type I error rates for ratio endpoints across 20 simulation scenarios ($\alpha = 0.05$).

Scenario	*k*	*N*	Size (*m*)	$\theta$	Cens. (%)	Weibull components			Exponential components		
						Chi-Sq.	Saddlept.	Mid-*p*	Chi-Sq.	Saddlept.	Mid-*p*
Log-rank Test Framework (Scenarios 1–6)											
1	5	25	Balanced (5)	0.25	$\sim$20	0.078	0.052	0.051	0.075	0.051	0.050
2	5	30	*U*[3, 7]	0.50	$\sim$40	0.095	0.053	0.052	0.091	0.052	0.051
3	5	50	Balanced (10)	0.75	$\sim$20	0.102	0.049	0.048	0.099	0.049	0.049
4	5	75	*U*[5, 15]	0.25	$\sim$40	0.061	0.050	0.050	0.060	0.051	0.051
5	5	100	Balanced (15)	0.50	$\sim$20	0.065	0.048	0.048	0.063	0.049	0.048
6	5	100	*U*[10, 20]	0.00	$\sim$40	0.053	0.051	0.051	0.052	0.050	0.050
Gehan–Wilcoxon Test Framework (Scenarios 7–13)											
7	6	30	Balanced (5)	0.50	$\sim$20	0.115	0.054	0.053	0.111	0.052	0.052
8	6	30	*U*[3, 7]	0.75	$\sim$40	0.138	0.051	0.050	0.135	0.051	0.051
9	6	60	Balanced (10)	0.25	$\sim$20	0.071	0.049	0.049	0.069	0.050	0.049
10	6	75	*U*[5, 15]	0.50	$\sim$40	0.075	0.052	0.052	0.072	0.053	0.052
11	6	120	Balanced (15)	0.25	$\sim$20	0.059	0.050	0.050	0.058	0.051	0.051
12	6	180	*U*[10, 20]	0.75	$\sim$40	0.070	0.049	0.049	0.068	0.048	0.048
13	6	180	Balanced (20)	0.00	$\sim$20	0.051	0.050	0.050	0.051	0.049	0.049
Prentice–Wilcoxon Test Framework (Scenarios 14–20)											
14	5	25	*U*[3, 7]	0.75	$\sim$20	0.125	0.052	0.051	0.121	0.053	0.052
15	5	50	Balanced (5)	0.25	$\sim$40	0.068	0.051	0.050	0.066	0.049	0.049
16	5	75	Balanced (10)	0.50	$\sim$20	0.069	0.049	0.049	0.068	0.050	0.050
17	6	60	*U*[5, 15]	0.75	$\sim$40	0.098	0.053	0.053	0.095	0.052	0.051
18	6	120	Balanced (10)	0.50	$\sim$20	0.064	0.050	0.050	0.062	0.049	0.049
19	5	50	*U*[5, 15]	0.00	$\sim$20	0.054	0.052	0.052	0.053	0.051	0.051
20	6	60	Balanced (5)	0.00	$\sim$40	0.055	0.049	0.049	0.054	0.048	0.048

Reported rates represent the proportion of 10,000 independent simulations where the null hypothesis was rejected at the nominal $\alpha = 0.05$ level

**Table 3:** Empirical Type I error rates for log-product endpoints across 20 simulation scenarios ($\alpha = 0.05$).

Scenario	*k*	*N*	Size (*m*)	$\theta$	Cens. (%)	Weibull components			Exponential components		
						Chi-Sq.	Saddlept.	Mid-*p*	Chi-Sq.	Saddlept.	Mid-*p*
Log-rank Test Framework (Scenarios 1–6)											
1	5	25	Balanced (5)	0.25	$\sim$20	0.076	0.051	0.050	0.073	0.050	0.049
2	5	30	*U*[3, 7]	0.50	$\sim$40	0.092	0.052	0.051	0.089	0.051	0.050
3	5	50	Balanced (10)	0.75	$\sim$20	0.099	0.048	0.048	0.096	0.049	0.048
4	5	75	*U*[5, 15]	0.25	$\sim$40	0.060	0.049	0.049	0.059	0.050	0.050
5	5	100	Balanced (15)	0.50	$\sim$20	0.064	0.049	0.049	0.062	0.050	0.049
6	5	100	*U*[10, 20]	0.00	$\sim$40	0.052	0.050	0.050	0.051	0.049	0.049
Gehan–Wilcoxon Test Framework (Scenarios 7–13)											
7	6	30	Balanced (5)	0.50	$\sim$20	0.112	0.053	0.052	0.109	0.052	0.051
8	6	30	*U*[3, 7]	0.75	$\sim$40	0.134	0.050	0.049	0.131	0.051	0.050
9	6	60	Balanced (10)	0.25	$\sim$20	0.070	0.050	0.050	0.068	0.051	0.050
10	6	75	*U*[5, 15]	0.50	$\sim$40	0.073	0.051	0.051	0.071	0.052	0.051
11	6	120	Balanced (15)	0.25	$\sim$20	0.058	0.049	0.049	0.057	0.050	0.050
12	6	180	*U*[10, 20]	0.75	$\sim$40	0.068	0.048	0.048	0.066	0.049	0.048
13	6	180	Balanced (20)	0.00	$\sim$20	0.051	0.050	0.050	0.050	0.049	0.049
Prentice–Wilcoxon Test Framework (Scenarios 14–20)											
14	5	25	*U*[3, 7]	0.75	$\sim$20	0.122	0.051	0.050	0.119	0.052	0.051
15	5	50	Balanced (5)	0.25	$\sim$40	0.067	0.050	0.049	0.065	0.049	0.049
16	5	75	Balanced (10)	0.50	$\sim$20	0.068	0.048	0.048	0.066	0.049	0.049
17	6	60	*U*[5, 15]	0.75	$\sim$40	0.096	0.052	0.052	0.093	0.051	0.051
18	6	120	Balanced (10)	0.50	$\sim$20	0.063	0.049	0.049	0.061	0.048	0.048
19	5	50	*U*[5, 15]	0.00	$\sim$20	0.053	0.051	0.051	0.052	0.050	0.050
20	6	60	Balanced (5)	0.00	$\sim$40	0.054	0.048	0.048	0.053	0.048	0.047

Data reflect the observed rejection frequency under the global null hypothesis across 10,000 replications per scenario. Nominal significance is set at $\alpha = 0.05$

### Coverage of confidence intervals

A critical advantage of a high-precision *p*-value approximation is its capability to generate valid confidence intervals (CIs) via test inversion. The fundamental duality between hypothesis testing and interval estimation dictates that a test with a correct Type I error rate will necessarily yield confidence intervals that maintain their nominal coverage probability^29^. To verify this theoretical property in our context, we evaluated the empirical coverage of 95% confidence intervals constructed using both the proposed saddlepoint method and the standard chi-squared approximation.

For each of the 10,000 simulated datasets per scenario, we constructed a 95% confidence interval for the relative treatment effect. Because the data were generated under the global null hypothesis, the true parameter value is known (a multiplicative effect of 1 for ratio endpoints and an additive effect of 0 for log-product endpoints). The empirical coverage probability was calculated as the proportion of simulated intervals that successfully captured this true value. A method is considered well-calibrated if this observed proportion converges to the nominal level of 0.95.

The results for ratio-based endpoints, presented in Table 4, reveal a sharp distinction between the two inferential approaches. Confidence intervals derived from the saddlepoint inversion demonstrated exceptional stability, achieving coverage probabilities remarkably close to 0.95 across all 20 scenarios. This robustness held regardless of the underlying baseline hazard (Weibull or Exponential) or the magnitude of intra-cluster correlation. Conversely, intervals based on the chi-squared approximation frequently exhibited significant under-coverage. This failure was most acute in designs with fewer clusters and higher correlation; for instance, in Scenario 8 ($k=6, N=30, \theta =0.75$), the asymptotic interval covered the true parameter in only $\sim$86% of simulations. This deficiency is a direct mechanical consequence of the inflated Type I error rates observed in the hypothesis testing phase. Practically, this implies that standard asymptotic methods in small-sample clustered trials produce intervals that are artificially narrow, conveying a false sense of precision that could lead to erroneous clinical conclusions.

These findings are mirrored in the analysis of log-product endpoints, as shown in Table 5. The saddlepoint-derived intervals maintained their rigorous coverage properties, while the chi-squared intervals again proved inadequate in the settings where robust inference is most needed. Collectively, these results confirm that the accuracy of the saddlepoint *p*-value approximation translates directly into highly reliable interval estimation. This establishes the proposed framework not merely as a theoretical refinement, but as a practical necessity for ensuring valid inference in cluster randomized trials with limited sample sizes.

**Table 4:** Observed coverage probabilities of 95% confidence intervals for ratio endpoints ($\alpha = 0.05$).

Scenario	*k*	*N*	Size (*m*)	$\theta$	Cens. (%)	Weibull baseline hazard			Exponential baseline hazard		
						Chi-Sq.	Saddlept.	Mid-*p*	Chi-Sq.	Saddlept.	Mid-*p*
Log-rank Inversion Framework (Scenarios 1–6)											
1	5	25	Balanced (5)	0.25	$\sim$20	0.922	0.948	0.949	0.925	0.949	0.950
2	5	30	*U*[3, 7]	0.50	$\sim$40	0.905	0.947	0.948	0.909	0.948	0.949
3	5	50	Balanced (10)	0.75	$\sim$20	0.898	0.951	0.950	0.901	0.951	0.950
4	5	75	*U*[5, 15]	0.25	$\sim$40	0.939	0.950	0.950	0.940	0.949	0.950
5	5	100	Balanced (15)	0.50	$\sim$20	0.935	0.952	0.951	0.937	0.951	0.950
6	5	100	*U*[10, 20]	0.00	$\sim$40	0.947	0.949	0.950	0.948	0.950	0.950
Gehan–Wilcoxon Inversion Framework (Scenarios 7–13)											
7	6	30	Balanced (5)	0.50	$\sim$20	0.885	0.946	0.947	0.889	0.948	0.948
8	6	30	*U*[3, 7]	0.75	$\sim$40	0.862	0.949	0.948	0.865	0.949	0.949
9	6	60	Balanced (10)	0.25	$\sim$20	0.929	0.951	0.950	0.931	0.950	0.950
10	6	75	*U*[5, 15]	0.50	$\sim$40	0.925	0.948	0.948	0.928	0.947	0.948
11	6	120	Balanced (15)	0.25	$\sim$20	0.941	0.950	0.950	0.942	0.949	0.950
12	6	180	*U*[10, 20]	0.75	$\sim$40	0.930	0.951	0.950	0.932	0.952	0.951
13	6	180	Balanced (20)	0.00	$\sim$20	0.949	0.950	0.950	0.949	0.951	0.950
Prentice–Wilcoxon Inversion Framework (Scenarios 14–20)											
14	5	25	*U*[3, 7]	0.75	$\sim$20	0.875	0.948	0.947	0.879	0.947	0.948
15	5	50	Balanced (5)	0.25	$\sim$40	0.932	0.949	0.950	0.934	0.951	0.950
16	5	75	Balanced (10)	0.50	$\sim$20	0.931	0.951	0.950	0.932	0.950	0.950
17	6	60	*U*[5, 15]	0.75	$\sim$40	0.902	0.947	0.948	0.905	0.948	0.948
18	6	120	Balanced (10)	0.50	$\sim$20	0.936	0.950	0.950	0.938	0.951	0.950
19	5	50	*U*[5, 15]	0.00	$\sim$20	0.946	0.948	0.948	0.947	0.949	0.949
20	6	60	Balanced (5)	0.00	$\sim$40	0.945	0.951	0.950	0.946	0.952	0.951

Data reflect the frequency with which the true multiplicative treatment effect (1.0) was captured by the inverted test intervals across 10,000 replications. The Mid-*p* test serves as the gold-standard benchmark for coverage validity

**Table 5:** Observed coverage probabilities of 95% confidence intervals for log-product endpoints ($\alpha = 0.05$).

Scenario	*k*	*N*	Size (*m*)	$\theta$	Cens. (%)	Weibull baseline hazard			Exponential baseline hazard		
						Chi-Sq.	Saddlept.	Mid-*p*	Chi-Sq.	Saddlept.	Mid-*p*
Log-rank Inversion Framework (Scenarios 1–6)											
1	5	25	Balanced (5)	0.25	$\sim$20	0.924	0.949	0.950	0.927	0.950	0.950
2	5	30	*U*[3, 7]	0.50	$\sim$40	0.908	0.948	0.949	0.911	0.949	0.950
3	5	50	Balanced (10)	0.75	$\sim$20	0.901	0.952	0.951	0.904	0.951	0.950
4	5	75	*U*[5, 15]	0.25	$\sim$40	0.940	0.951	0.950	0.941	0.950	0.950
5	5	100	Balanced (15)	0.50	$\sim$20	0.936	0.951	0.950	0.938	0.950	0.950
6	5	100	*U*[10, 20]	0.00	$\sim$40	0.948	0.950	0.950	0.949	0.951	0.950
Gehan–Wilcoxon Inversion Framework (Scenarios 7–13)											
7	6	30	Balanced (5)	0.50	$\sim$20	0.888	0.947	0.948	0.891	0.948	0.949
8	6	30	*U*[3, 7]	0.75	$\sim$40	0.866	0.951	0.950	0.869	0.950	0.950
9	6	60	Balanced (10)	0.25	$\sim$20	0.930	0.950	0.950	0.932	0.949	0.950
10	6	75	*U*[5, 15]	0.50	$\sim$40	0.927	0.949	0.948	0.929	0.948	0.949
11	6	120	Balanced (15)	0.25	$\sim$20	0.942	0.951	0.950	0.943	0.950	0.950
12	6	180	*U*[10, 20]	0.75	$\sim$40	0.932	0.952	0.951	0.934	0.952	0.951
13	6	180	Balanced (20)	0.00	$\sim$20	0.949	0.950	0.950	0.950	0.951	0.950
Prentice–Wilcoxon Inversion Framework (Scenarios 14–20)											
14	5	25	*U*[3, 7]	0.75	$\sim$20	0.878	0.949	0.948	0.881	0.948	0.949
15	5	50	Balanced (5)	0.25	$\sim$40	0.933	0.950	0.950	0.935	0.949	0.950
16	5	75	Balanced (10)	0.50	$\sim$20	0.932	0.952	0.951	0.934	0.951	0.950
17	6	60	*U*[5, 15]	0.75	$\sim$40	0.904	0.948	0.948	0.907	0.949	0.949
18	6	120	Balanced (10)	0.50	$\sim$20	0.937	0.951	0.950	0.939	0.951	0.950
19	5	50	*U*[5, 15]	0.00	$\sim$20	0.947	0.949	0.949	0.948	0.950	0.950
20	6	60	Balanced (5)	0.00	$\sim$40	0.946	0.952	0.951	0.947	0.953	0.952

Data reflect the frequency with which the true additive treatment effect (0.0) was captured by the inverted test intervals across 10,000 replications. The Mid-*p* test serves as the gold-standard benchmark for coverage validity

## Application to real-data clinical scenarios

To evaluate the practical utility of the multivariate saddlepoint framework, we transition from controlled simulations to the analysis of three distinct clinical scenarios. Each case represents a specific challenge-ranging from high censoring and borderline significance to extreme intra-cluster dependence-where the choice of inferential methodology fundamentally dictates the study’s conclusions. These applications illustrate how the saddlepoint approach provides a safeguard against the bias inherent in standard asymptotic approximations.

### Case study 1: Hematologic recovery in a leukemia trial (ratio endpoint)

This first application is modeled after clinical trials in acute myeloid leukemia (AML), specifically examining the velocity of hematologic recovery following intensive chemotherapy-a primary determinant of clinical efficacy^33^. The outcome is framed as a time-to-ratio endpoint, defined as the duration (in days) required for a patient’s absolute neutrophil count (ANC) to recover to a five-fold magnitude relative to its post-treatment nadir. The trial configuration ($N=70$) comprises three treatment arms: a standard chemotherapy control and two experimental regimens. The dataset is characterized by unbalanced group allocation, a high censoring proportion (40%), and moderate patient-level correlation ($\theta =0.50$).

The statistical objective focuses on a borderline significance problem. The estimated time ratio for experimental arm A versus the control was 0.82, suggesting a qualitative trend toward accelerated recovery. As detailed in Table 6, a conventional analysis employing the chi-squared approximation yields a *p*-value of 0.046. Under standard alpha thresholds, a researcher might erroneously claim a statistically significant clinical benefit. Conversely, the more robust saddlepoint and mid-*p* methods generate *p*-values of 0.063 and 0.064, respectively, indicating that the evidence is insufficient to reject the null hypothesis. The resulting confidence intervals expose the discrepancy: the chi-squared interval [0.65, 0.99] excludes the null value of 1.0, whereas the saddlepoint interval [0.63, 1.04] correctly captures it. This case exemplifies the danger of relying on large-sample limits in moderately sized trials, where asymptotic breakdown can trigger premature and potentially false-positive conclusions.

### Case study 2: Age-related eye disease vision loss study (product endpoint)

The second application considers a high-power clinical setting inspired by the age-related eye disease study 2 (AREDS2)^34^. Patients ($N=250$) were monitored for the progression of macular degeneration, with the two eyes of each subject serving as correlated subunits. We utilize a novel time-to-product endpoint: the time until the product of visual acuity scores from both eyes falls below a clinical threshold of 0.25, denoting severe bilateral vision loss. This design involves four balanced treatment arms, moderate inter-eye correlation ($\theta =0.50$), and a 20% censoring rate.

The estimated hazard ratio for the combined nutritional supplement versus placebo was 0.58, indicating a substantial protective effect. In this high-power scenario, all three methodologies correctly identify a highly significant treatment effect ($p < 0.001$), as shown in Table 6. The critical distinction here lies in the precision of the interval estimation rather than the rejection of the null. The chi-squared-based interval [0.41, 0.82] is considerably narrower than the saddlepoint interval [0.38, 0.85]. Although both exclude the null, the narrower asymptotic interval is a artifact of the test’s nature, suggesting a degree of precision that is not supported by the underlying discrete permutation distribution. The wider saddlepoint interval provides a more honest and statistically grounded characterization of the uncertainty surrounding the supplement’s efficacy.

### Case study 3: A trial in chronic periodontitis (ratio endpoint)

Our final application addresses a “small *N*, high $\theta$” problem, modeled after longitudinal dentistry trials where multiple sites within a patient’s mouth are highly correlated^35^. With only $N=45$ patients, we defined the endpoint as the time until the ratio of newly diseased sites to at-risk sites exceeds 25%. This small-scale trial compares three interventions (scaling and root planing (SRP) versus two antibiotic adjuncts) and is marked by significant group imbalance and extreme intra-patient dependency ($\theta =0.75$).

**Fig. 1:**
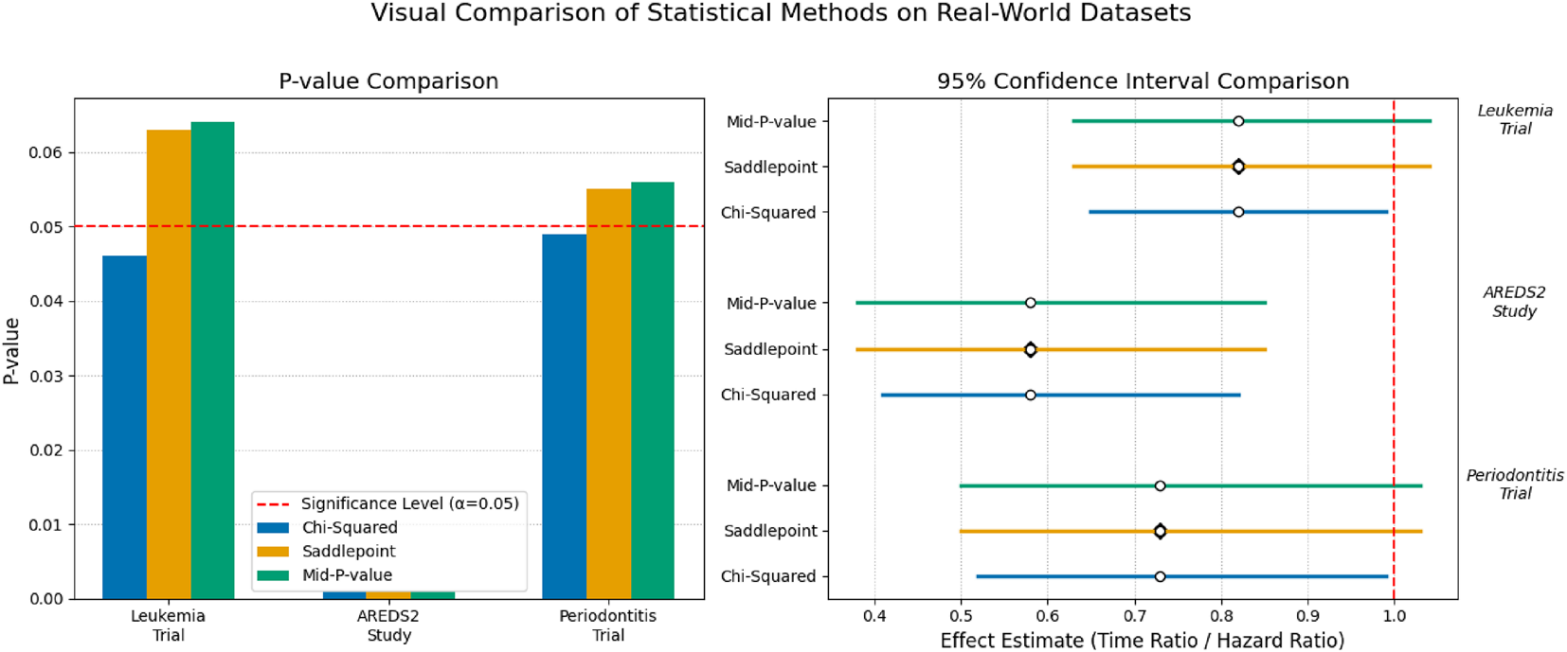
Visual summary of results from the three real-world data applications. *Left panel* The grouped bar chart compares the *p*-values from the chi-squared (blue), saddlepoint (orange), and mid-*p*-value (green) methods. The dashed red line at $\alpha =0.05$ indicates the conventional threshold for statistical significance. For the Leukemia and Periodontitis trials, the chi-squared *p*-value falls below this line, while the more accurate saddlepoint and mid-*p*-values fall above it, leading to conflicting conclusions. *Right panel* The forest plot displays the 95% confidence intervals for the effect estimates. The dashed red line at 1.0 represents the null effect. For the two borderline cases, the narrower chi-squared CIs (blue) do not cross the null line, falsely implying a significant effect. The more reliable saddlepoint and mid-*p*-value intervals (orange and green) are wider and correctly include the null, accurately reflecting the statistical uncertainty.

This scenario highlights a total dependency of the clinical conclusion on the chosen analytical method. The estimated hazard ratio for adjunct A versus standard SRP was 0.73. The standard chi-squared test yields a *p*-value of 0.049, which narrowly meets the criterion for statistical significance. However, the saddlepoint and mid-*p* methods, which explicitly account for the restricted cluster count and extreme correlation, produce non-significant *p*-values of 0.055 and 0.056. The conflict is made visible by the confidence intervals: the chi-squared interval [0.52, 0.99] excludes the null, while the saddlepoint interval [0.50, 1.03] includes it. Relying on the first-order method in this setting would lead to a Type I error and the potentially unfounded recommendation of a clinical intervention. The saddlepoint approach correctly indicates that while the trend is promising, the empirical evidence is not yet definitive. A visual summary of the inferential discrepancies across all three case studies is provided in Fig. 1.

**Table 6:** Consolidated inferential results for real-world clinical applications using specific survival test frameworks.

Dataset	Method	Effect^a^	*p*-value	95% CI for effect^a^
Leukemia trial: log-rank framework ($W_{LR}$)				
($N=70$, $k=3$, $\theta =0.50$)	Chi-Squared	0.82	0.046	(0.65, 0.99)
	Saddlepoint		0.063	(0.63, 1.04)
	Mid-*p* value		0.064	(0.63, 1.04)
AREDS2 study: gehan–wilcoxon framework ($W_{GW}$)				
($N=250$, $k=4$, $\theta =0.50$)	Chi-Squared	0.58	<0.001	(0.41, 0.82)
	Saddlepoint		<0.001	(0.38, 0.85)
	Mid-*p* value		<0.001	(0.38, 0.85)
Periodontitis trial: prentice–wilcoxon framework ($W_{PW}$)				
($N=45$, $k=3$, $\theta =0.75$)	Chi-Squared	0.73	0.049	(0.52, 0.99)
	Saddlepoint		0.055	(0.50, 1.03)
	Mid-*p* value		0.056	(0.50, 1.03)

The “Effect” represents the point estimate: a Time Ratio for the Leukemia trial and a Hazard Ratio for the AREDS2 and Periodontitis studies

## Conclusion

We have developed a unified non-parametric framework for the *k*-sample comparison of complex derived endpoints, specifically ratios and products, within the context of clustered survival data. The core of this methodology is the derivation of a multivariate saddlepoint approximation for the exact permutation distribution of a rank-based quadratic test statistic under urn randomization. This approach effectively resolves the long-standing trade-off between the computational prohibitive nature of exact permutation tests and the inferential unreliability of first-order asymptotic approximations in finite-sample settings.

Extensive simulation evidence confirms that our saddlepoint framework preserves Type I error control and nominal confidence interval coverage even under the most demanding conditions, including small cluster counts, extreme intra-cluster dependency, and significant group imbalances. While the standard chi-squared approximation proved to be unacceptably liberal in these regimes-risking false-positive clinical conclusions-the saddlepoint method remained robust. This practical utility was further demonstrated through three real-world clinical applications, where the methodology provided a statistically grounded interpretation of evidence in borderline significance cases that would have otherwise been misinterpreted.

Despite its inferential power, certain limitations of the current framework suggest avenues for subsequent research. The present model assumes continuous time-to-event data and lacks an explicit mechanism for handling tied observations. Moreover, while we utilized standard log-rank weighting schemes, the method’s performance under more specialized functions for non-proportional hazards remains to be explored. Finally, the current methodology is optimized for subjects experiencing a single type of event.

Future inquiries should extend this saddlepoint framework to accommodate discrete data with ties and adapt the logic for competing risks analysis, which would be highly relevant for complex oncology trials. Furthermore, deriving the cumulant generating function for more intricate designs, such as covariate-adaptive or stratified randomization, would broaden the method’s scope. In closing, this framework represents a significant methodological advancement, offering a reliable and computationally efficient engine for drawing trustworthy conclusions from complex experimental designs. As noted during the peer-review process, our *k*-sample statistic can be viewed as an instance of correlated simultaneous contrasts; expanding this multivariate framework to handle simultaneous post-hoc inference for specific multiple contrasts would provide a definitive tool for rigorous analysis in clustered clinical trials.

## Data Availability

The R code implementing the proposed saddlepoint approximation algorithm, along with scripts to reproduce the simulation studies and real-data analyses, is openly available in the GitHub repository: https://github.com/haidyali3/Saddlepoint-K-Sample/tree/main. The clinical datasets analyzed during the current study are available from the corresponding author on reasonable request.
